# Supplementary material for: Effectiveness of lumbar support with built-in massager system on spinal angle profiles among high-powered traffic police motorcycle riders: A randomised controlled trial
Source: PLoS One. 2021 Oct 19;16(10):e0258796. doi: 10.1371/journal.pone.0258796 (PMC8525748; doi:10.1371/journal.pone.0258796)
Supplement: S1 Table — (DOCX) [file pone.0258796.s001.docx]

**S1 Table. Spinal Angle Profile Throughout 20 Minutes (N=24)**

| **Sensor** | **Group** | **(Reference Angle (°))** | **Riding duration (minutes)** | | | | | | | | | | |
| --- | --- | --- | --- | --- | --- | --- | --- | --- | --- | --- | --- | --- | --- |
|  |  |  | **2** | **4** | **6** | **8** | **10** | **12** | **14** | **16** | **18** | **20** |  |
| S1 | Control_P_0_ | **25.46** | **25.48** | **28.51** | **26.93** | **29.61** | **30.72** | **29.66** | **30.84** | **35.91** | **37.42** | **39.98** |  |
|  |  | Deviation | +0.02 | +3.05 | +1.47 | +4.15 | +5.26 | +4.20 | +5.38 | +10.45 | +11.96 | +14.52 |  |
|  | Control_P_1_ | **28.43** | **28.44** | **29.94** | **31.89** | **32.71** | **30.91** | **33.75** | **33.44** | **35.00** | **38.15** | **38.66** |  |
|  |  | Deviation | +0.01 | +1.51 | +3.46 | +4.28 | +2.48 | +5.32 | +5.01 | +6.57 | +9.72 | +10.23 |  |
|  | Experimental_P_0_ | **27.35** | **27.37** | **30.47** | **29.10** | **31.89** | **30.90** | **31.97** | **34.68** | **37.35** | **38.88** | **39.99** |  |
|  |  | Deviation | +0.02 | +3.12 | +1.75 | +4.54 | +3.55 | +4.62 | +7.33 | +10.00 | +11.53 | +12.64 |  |
|  | Experimental_P_1_ | **20.00** | **20.01** | **20.99** | **20.10** | **22.90** | **21.01** | **22.78** | **24.80** | **24.77** | **24.64** | **24.45** |  |
|  |  | Deviation | +0.01 | +0.99 | +0.10 | +2.90 | +1.01 | +2.78 | +4.80 | +4.77 | +4.64 | +4.45 |  |
| **S2** | Control_P_0_ | **3.99** | **4.07** | **7.64** | **5.23** | **8.59** | **9.04** | **8.62** | **9.10** | **10.84** | **12.06** | **13.93** |  |
|  |  | Deviation | +0.08 | +3.65 | +1.24 | +4.60 | +5.05 | +4.63 | +5.11 | +6.85 | +8.07 | +9.94 |  |
|  | Control_P_1_ | **4.42** | **4.44** | **8.01** | **8.63** | **8.56** | **8.25** | **9.27** | **9.11** | **10.07** | **12.76** | **12.90** |  |
|  |  | Deviation | +0.02 | +3.97 | +4.21 | +4.14 | +3.83 | +4.85 | +4.69 | +5.65 | +8.34 | +8.48 |  |
|  | Experimental_P_0_ | **4.33** | **4.32** | **7.48** | **6.92** | **8.60** | **8.47** | **8.92** | **10.22** | **11.93** | **12.55** | **13.57** |  |
|  |  | Deviation | +0.01 | +3.15 | +2.59 | +4.27 | +4.14 | +4.59 | +5.89 | +7.60 | +8.12 | +9.24 |  |
|  | Experimental_P_1_ | **3.05** | **3.07** | **3.98** | **3.70** | **4.93** | **4.06** | **4.21** | **7.55** | **5.43** | **6.30** | **6.23** |  |
|  |  | Deviation | +0.02 | +0.93 | +0.65 | +1.88 | +1.01 | +1.16 | +4.50 | +2.38 | +3.25 | +3.18 |  |
| **S3** | Control_P_0_ | **-12.44** | **-12.40** | **-10.34** | **-11.72** | **-9.80** | **-7.91** | **-7.18** | **-7.79** | **-5.76** | **-3.00** | **-2.57** |  |
|  |  | Deviation | +0.04 | +2.10 | +0.70 | +2.24 | +4.53 | +5.26 | +4.65 | +6.68 | +9.44 | +9.87 |  |
|  | Control_P_1_ | **-13.85** | **-13.82** | **-9.71** | **-9.03** | **-9.22** | **-9.54** | **-8.26** | **-8.32** | **-7.91** | **-5.08** | **-4.34** |  |
|  |  | Deviation | +0.03 | +4.14 | +4.82 | +4.63 | +4.31 | +5.59 | +5.53 | +5.94 | +8.77 | +9.51 |  |
|  | Experimental_P_0_ | **-13.75** | **-13.72** | **-10.64** | **-11.97** | **-9.75** | **-9.89** | **-9.96** | **-7.73** | **-6.44** | **-5.21** | **-4.03** |  |
|  |  | Deviation | +0.03 | +3.11 | +1.78 | +4.00 | +3.86 | +3.79 | +6.02 | +7.31 | +8.54 | +9.72 |  |
|  | Experimental_P_1_ | **-11.99** | **-11.97** | **-10.90** | **-11.45** | **-10.17** | **-10.01** | **-9.95** | **-9.46** | **-10.27** | **-10.66** | **-10.80** |  |
|  |  | Deviation | +0.02 | +1.09 | +0.54 | +1.82 | +1.98 | +2.04 | +2.53 | +1.72 | +1.33 | +1.19 |  |
| **S4** | Control_P_0_ | **-16.01** | **-15.98** | **-12.74** | **-14.20** | **-11.93** | **-9.87** | **-9.04** | **-8.92** | **-5.46** | **-3.32** | **-3.09** |  |
|  |  | Deviation | +0.03 | +3.27 | +1.80 | +4.08 | +6.14 | +6.97 | +7.09 | +10.55 | +12.69 | +12.92 |  |
|  | Control_P_1_ | **-16.93** | **-16.90** | **-12.54** | **-11.94** | **-12.05** | **-12.36** | **-10.94** | **-10.45** | **-9.13** | **-7.11** | **-6.26** |  |
|  |  | Deviation | +0.03 | +4.39 | +4.99 | +4.88 | +4.57 | +5.99 | +6.48 | +7.80 | +9.58 | +10.65 |  |
|  | Experimental_P_0_ | **-16.69** | **-16.54** | **-13.51** | **-14.89** | **-12.83** | **-12.98** | **-11.01** | **-8.74** | **-7.98** | **-6.39** | **-5.17** |  |
|  |  | Deviation | +0.15 | +3.18 | +1.80 | +3.86 | +3.71 | +5.68 | +7.95 | +8.71 | +10.30 | +11.52 |  |
|  | Experimental_P_1_ | **-15.43** | **-15.40** | **-14.32** | **-15.23** | **-14.99** | **-14.45** | **-13.96** | **-12.74** | **-14.73** | **-13.02** | **-13.92** |  |
|  |  | Deviation | +0.03 | +1.11 | +0.20 | +0.44 | +0.98 | +1.47 | +2.67 | +0.70 | +2.41 | +1.51 |  |
| **S5** | Control_P_0_ | **7.89** | **7.91** | **8.51** | **8.65** | **8.91** | **8.95** | **9.02** | **9.26** | **9.57** | **9.71** | **9.89** |  |
|  |  | Deviation | +0.02 | +0.62 | +0.76 | +1.02 | +1.06 | +1.13 | +1.37 | +1.68 | +1.82 | +2.00 |  |
|  | Control_P_1_ | **8.11** | **8.12** | **8.98** | **9.00** | **9.12** | **9.24** | **9.37** | **9.39** | **9.42** | **9.50** | **9.53** |  |
|  |  | Deviation | +0.01 | +0.87 | +1.11 | +1.01 | +1.13 | +1.26 | +1.28 | +1.31 | +1.39 | +1.42 |  |
|  | Experimental_P_0_ | **7.97** | **8.00** | **8.52** | **8.10** | **8.95** | **8.71** | **9.01** | **9.05** | **9.13** | **9.16** | **9.17** |  |
|  |  | Deviation | +0.03 | +0.55 | +0.13 | +0.98 | +0.74 | +1.04 | +1.08 | +1.16 | +1.19 | +1.20 |  |
|  | Experimental_P_1_ | **6.05** | **6.05** | **6.20** | **6.07** | **6.18** | **6.25** | **6.40** | **6.66** | **6.28** | **6.52** | **6.51** |  |
|  |  | Deviation | +0.00 | +0.15 | +0.02 | +0.13 | +0.20 | +0.35 | +0.61 | +0.23 | +0.47 | +0.46 |  |
