# Supplementary material for: Effectiveness of lumbar support with built-in massager system on spinal angle profiles among high-powered traffic police motorcycle riders: A randomised controlled trial
Source: PLoS One. 2021 Oct 19;16(10):e0258796. doi: 10.1371/journal.pone.0258796 (PMC8525748; doi:10.1371/journal.pone.0258796)
Supplement: Attachment — Submitted filename: Manuscript.docx [file pone.0258796.s002.docx]

**Assessment of spinal angle profiles among high-powered traffic police motorcycle riders**

Nur Athirah Diyana Mohammad Yusof^1¶^, Karmegam Karupiah*^1¶^, Shamsul Bahri Mohd Tamrin ^1&^, Irniza Rasdi^1^, Vivien How^1&^, Sivasankar Sambasivam^1&^, Putri Anis Syahira^1¶^, Kulanthayan K. C. Mani^2&^, Hassan Sadeghi Naeini^3&^, Dayana Hazwani Mohd Suadi Nata^4&^

^1^Department of Environmental and Occupational Health, Faculty of Medicine and Health Sciences, Universiti Putra Malaysia, UPM Serdang, Selangor, Malaysia

^2^Department of Community Health, Faculty of Medicine and Health Sciences, Universiti Putra Malaysia, UPM Serdang, Selangor, Malaysia

^3^Industrial Design Department, School of Architecture & Environmental Design, Iran University of Science & Technology, Tehran, Iran

^4^Faculty of Industrial Science and Technology, Universiti Malaysia Pahang, Lebuhraya Tun Razak, Gambang Kuantan Pahang, Malaysia

*Corresponding author

Email: [megam@upm.edu.my](mailto:megam@upm.edu.my)

^¶^These authors contributed equally to this work.

^&^These authors also contributed equally to this work.

**ABSTRACT**

The study aimed to assess and compare spinal angle profiles of traffic police riders using two types of motorcycle seat: existing motorcycle seat (without lumbar support) and prototype motorcycle seat (lumbar support with built-in massager system). An experimental study was conducted among 24 traffic police riders (12 riders each in the control and experimental groups) who participated in this study. Each participant was required to wear a TruPosture Smart Shirt (to monitor spinal posture) while riding their high-powered motorcycle for 20 minutes. The TruPosture Apps recorded the spinal angle posture pattern. The data indicated that the police riders using motorcycle seat with lumbar support and built-in massager system showed a huge improvement in maintaining posture which only involves slight spinal angle deviation changes from the spinal reference angle throughout the 20 minutes ride. A Wilcoxon signed-rank test revealed a statistically significant difference between pre-test and post-test session in all sensors at the 0th, 10th and 20th minutes in the experimental group (p<0.05). Meanwhile, no statistically significant difference is observed among the control group in all five sensors (p>0.05). An ergonomic intervention prototype (lumbar support with built-in massager system) successfully helps to maintain and improve the natural curve of the spinal posture. This indirectly would reduce musculoskeletal disorders and muscle discomfort among traffic police riders.

Keywords: posture, lumbar support, massage system, intervention, seat

**INTRODUCTION**

The riding posture is very important because awkward riding posture may cause discomfort to the riders and increase the risk of developing MSD in long-term exposure. De Cássia et al. [1] stated that body discomfort is an indicator of many musculoskeletal disorders due to prolonged discomfort. A sustained flexion of lumbar lordosis (low back) during driving usually is the main factor in the generation of low back pain [2]. To improve spinal posture and comfort in transportation settings, the seat design has a significant role and efficiency.

A well-designed seat would decrease pressure within the spinal discs, spinal ligaments, and gluteal muscle [3-6]. Lumbar support is the solution for the problem, whereby the weight and pressure of the trunk are taken by the back support and increased lumbar lordosis. The use of a vehicle seat (lumbar support) has seen significantly associated with the reduction of muscle discomfort and low back pain during prolonged riding and driving journey [3,6]. Adjustable lumbar support with extra cushioning, which provided a massage-like comfort, have been proved to reduce transmission of vibration, which indirectly increases muscle comfort by improving oxygen and blood flow to the tissue [7,8]. According to Mansfield et al. [9], the seat shape, user suitability, seat material, duration of sitting in the same position, vibration, and posture changes are among the factors that influence seat comfort.

Traffic police is a police officer who serves to enforce the rules related to traffic. They must perform various work tasks, such as escorting the important person in any events, patrol selected locations, and finding any road offenders. All this work task requires them to use a motorcycle as their vehicle. At present, the officer riding a motorcycle takes at least 5 hours per day during duties. Therefore, traffic police riders have to endure prolonged riding duration. Rashid et al. [10] believed that continuous riding of a standard motorcycle for an extended time would result in a high level of postural fatigue and health problems. However, to the best of the authors' knowledge, no research has been done on the spinal riding posture during a motorcycle ride, especially among police officers in Malaysia who use a motorcycle to perform their duties daily.

To solve this problem, ergonomics is the best solution for scientific research in man-machine interaction at the workplace since this field involves fitting machine to workers comfortability to improve their working performance, reduce fatigue, and stress [11]. The ergonomic application is very significant in areas involving prolonged and static riding activities that directly affect the riders' healthy spinal posture and reduce muscle fatigue. Thus, this research aims to compare spinal angle profiles of traffic police riders between pre-test and post-test studies in two groups (control and experimental).

**MATERIALS AND METHODS**

**Subjects**

An experimental study was conducted among 24 traffic policemen who ride a high-powered motorcycle (Honda CBX 750), with no history of MSD and low back pain. The subjects were recruited with a mean age of 29.6 ± 4.79 years, a height of 1.73 ± 0.06 cm, and a weight of 72.7 ± 9.97 kg. Most of the respondents served as traffic police officers for more than five years and above.

**A prototype**

The lumbar support on the prototype seat can be adjusted either upward and downward according to the lumbar height position for the comfortability of the riders. There is also a massage fitted inside the lumbar support with two rotating balls. The lumbar massage was set at a ratio of 1 minute on and 4 minutes off. It was used for 20 minutes in the experimental riding session. It can support up to 150 kg body weight.

**TruPosture**

A spinal posture angle was obtained using the TruPosture Smart Shirt connected with the TruPosture mobile app among the police riders while riding a motorcycle with existing seat for both groups; control and experimental during the pre-test. Then, during the post-test, the control group used the same motorcycle seat, and the experimental group used a prototype seat containing adjustable lumbar support with a build-in massager system. The TruPosture Smart Shirt covered different regions of the spinal alignment, with five nano-sensor technology attached undershirt. The five sensors cover the thoracic vertebrae, lumbar, and pelvic regions (T1, T8, L1, L3, and Pelvis).

The technology helped the researcher monitor the entire spinal’s alignment and curvature in several modes, including standing and sitting. The TruPosture mobile apps interface (Fig 1) and Windows software were utilised as these tools help the researcher track the posture and record the movement of posture in real-time. The sensors detect the real-time changes of spinal posture angle based on the spinal movement.

**Fig 1.** **TruPosture Mobile Apps Interface**.

Based on Fig 1, the blue curve represents the spinal reference posture (ideal posture) at 0 minutes, and the orange curve indicates the actual spinal riding posture throughout 20 minutes. The number on the left and right side of the interface represents the angle reading for each spinal point sensor. The positive and negative values of angle indicate the spinal position, which is forward and backward.

**Data Collection**

In this study, the spinal reference posture at the 0 minutes riding session was set first. The respondents wore the TruPosture Smart Shirt during the monitoring, according to the ideal riding posture. An upright riding posture is the best posture in riding this type of motorcycle based on the standard operating procedure (SOP) of Honda CBX750P motorcycle riding. According to Ma’arof et al. [12], a good riding posture for the upright posture is that the arms should bend slightly when gripping the throttle and sitting in the upright position. The shoulders and elbows are held easily on the holds without exceeding or over-expanding the elbows. Elbows are flexed, and lower arms are parallel to the ground. Furthermore, the legs are held near the fuel tank while relaxing the hip and pelvis. This posture was set as a spinal reference posture for riding, and the angle for each sensor was recorded. However, this is the real problem for the riders, as they could not maintain the ideal posture in medium- and long-distance trips.

This study was conducted at the MEX highway route (the route between the commercial centre of Kuala Lumpur to the Federal Administrative capital of Putrajaya and Kuala Lumpur International Airport), with good road condition and maintenance. The route was chosen as it was commonly used in their work task as escort riders. The pre-post test results were then compared between the two groups. The summary of the experimental study flow is illustrated in Fig 2.

**Fig 2. A summary of the experimental study flow**

**Statistical Analysis**

The collected data from the questionnaire and measurement were analysed using the IBM SPSS Software (Version 26). The data collected were analysed using univariate and bivariate analyses, while the Wilcoxon signed-ranked test was used to verify a statistically significant difference between the control and experimental groups. The study was conducted using a 95% confidence level, 80% of power, and the results of p≤0.05 were considered significant.

**Ethics Approval**

This study was submitted and approved by the Ethics Committee, Universiti Putra Malaysia (reference number: UPM/TNCPI/RMC/JKEUPM/1.4.18.2 (JKEUPM)). Permission from the respondents of this study was obtained with their written consents before the study was conducted. Privacy of the information and confidentiality of the respondents were and are always protected.

**RESULTS**

**Distribution of five different sensors for spinal posture angle**

The mean spinal posture angle in each sensor for the pre-test and post-test sessions between the two groups is presented in Fig 3. The graph showed that the experimental post-test values are successfully maintained with minimal changes to the spinal posture angle compared to the other groups. The positive and negative values of angle indicate the position of the spinal, which is forward and backwards, respectively.

**Fig 3. Mean spinal posture angle changes throughout 20 minutes riding for pre-test and post-test study.** C_P_0_: a control pre-test group; C_P_1_: a control post-test; E­_P_0_: Experimental pre-test; E_P_1_: Experimental post test

Meanwhile, the line graph in Fig 4 (a–e) show the trend comparison of spinal posture angle deviation throughout the 20 minutes riding session. The line graph compares the level of spinal angle deviation in four groups (control pre-test, experimental pre-test, control post-test, and experimental post-test). Based on the graph line comparison, a deviation of spinal angle increased in each sensor, and Sensor 1 experienced the greatest deviation than the other sensors. From the graph, a deviation of spinal angle on each group shows an upward trend. However, the spinal angle deviation in the experimental post-test group is always at a lower level than the other groups. It showed that the experimental group intervention has a huge improvement in maintaining posture, which only involves slight spinal angle deviation changes from the spinal reference angle throughout the 20 minutes ride. Overall, the highest peak of spinal angle deviation from the graph is at the end of the experimental session for each sensor.

**Fig 4. (a–e). Deviation of spinal posture angle throughout 20 minutes (N=24)**

Sensor 1 has the highest deviation in the control pre-test (+14.52) at the 20^th^ minute, followed by Sensor 4 in the control pre-test (+12.92, +12.69) at the 20^th^ and 18^th^ minute throughout the measurement. The details of the spinal posture angle profile deviation throughout 20 minutes are tabulated in Table 1.

**Table 1. The minimum and maximum value of a deviation spinal angle profile throughout 20 minutes (N=24)**

| **Sensor** | **Deviation from Reference Spinal Angle (Min^o^-Max^o^)** | | | |
| --- | --- | --- | --- | --- |
|  | **0-20 minutes** | | | |
|  | **Control_P_0_** | **Control_P_1_** | **Experimental_P_0_** | **Experimental_P_1_** |
| S1 | 0.02-14.52 | 0.01-10.23 | 0.02-12.64 | 0.01-5.71 |
| S2 | 0.08-9.94 | 0.02-8.48 | 0.01-9.24 | 0.02-4.50 |
| S3 | 0.04-9.87 | 0.03- 9.51 | 0.03- 9.72 | 0.02-2.53 |
| S4 | 0.03-12.92 | 0.03-10.65 | 0.15-11.52 | 0.03-2.67 |
| S5 | 0.02-2.00 | 0.01-1.42 | 0.03-1.20 | 0.00-0.61 |

**Comparison of Five Different Sensors Between Pre-Test and Post-Test Studies**

Table 2 compares the spinal angle (0^th^, 10^th^, and 20^th^ minutes) between pre-test and post-test study for five different sensors. As depicted in this table, a Wilcoxon signed-rank test revealed a statistically significant difference between pre-test and post-test session in all sensors at the 0^th^, 10^th^ and 20^th^ minutes in the experimental group (p<0.05). Meanwhile, no statistically significant difference is observed among the control group in all five sensors (p>0.05).

**Table 2. A median score of five different sensors between pre-test and post-test studies**

| **Sensor** | **Group** | **Minute** | **Median (IQR)** | | **Z-statistics** | **p-value** |
| --- | --- | --- | --- | --- | --- | --- |
|  |  |  | **Pre-** | **Post-** |  |  |
| 1 | Control | 0 | 25.5 (2.54) | 28.4 (2.57) | -1.193 | 0.471 |
|  | Experimental |  | 25.7 (2.75) | 20.5 (3.75) | -3.065 | 0.002** |
|  | Control | 10 | 30.7(3.50) | 30.9 (1.21) | -0.471 | 0.638 |
|  | Experimental |  | 30.7 (1.75) | 21.0 (1.76) | -3.063 | 0.002** |
|  | Control | 20 | 38.5 (2.73) | 38.6 (1.76) | -1.021 | 0.307 |
|  | Experimental |  | 40.0 (1.75) | 24.5 (1.00) | -3.070 | 0.002** |
| **2** | Control | 0 | 4.0 (2.50) | 4.71 (2.00) | -1.615 | 0.106 |
|  | Experimental |  | 4.8 (1.75) | 3.71 (1.00) | -2.914 | 0.004** |
|  | Control | 10 | 9.0 (1.75) | 8.13 (1.75) | -0.566 | 0.572 |
|  | Experimental |  | 8.5 (1.60) | 4.06 (0.94) | -3.063 | 0.002** |
|  | Control | 20 | 12.5 (2.70) | 12.0 (3.00) | -1.725 | 0.084 |
|  | Experimental |  | 13.6 (0.32) | 6.23 (0.98) | -3.061 | 0.002** |
| **3** | Control | 0 | -12.0 (2.75) | -11.0 (3.00) | -0.751 | 0.453 |
|  | Experimental |  | -13.0 (2.69) | -11.0 (1.75) | -2.754 | 0.006** |
|  | Control | 10 | -7.0 (1.75) | -7.5 (2.75) | -1.561 | 0.119 |
|  | Experimental |  | -8.0 (2.75) | -9.0 (2.75) | -2.673 | 0.008** |
|  | Control | 20 | -3.0 (1.00) | -3.5 (1.00) | -1.308 | 0.191 |
|  | Experimental |  | -4.00 (1.75) | -10.5 (1.00) | -3.077 | 0.002** |
| **4** | Control | 0 | -16.0 (2.50) | -16.0 (1.75) | -0.206 | 0.837 |
|  | Experimental |  | -16.0 (2.00) | -15.0 (1.50) | -2.263 | 0.024* |
|  | Control | 10 | -10.0 (1.75) | -11.5 (2.75) | -1.807 | 0.071 |
|  | Experimental |  | -12.0 (1.75) | -14.0 (1.75) | -2.792 | 0.037* |
|  | Control | 20 | -4.0 (2.00) | -4.5 (2.75) | -1.083 | 0.279 |
|  | Experimental |  | -5.0 (1.75) | -12.0 (2.00) | -3.074 | 0.002* |
| **5** | Control | 0 | 7.0 (1.75) | 8.0 (2.75) | -1.533 | 0.125 |
|  | Experimental |  | 8.0 (1.75) | 6.0 (1.75) | -2.090 | 0.037* |
|  | Control | 10 | 8.0 (2.00) | 9.0 (2.00) | -1.491 | 0.136 |
|  | Experimental |  | 9.0 (1.75) | 6.0 (1.75) | -3.129 | 0.002** |
|  | Control | 20 | 9.0 (1.00) | 10.0 (2.0) | -1.408 | 0.159 |
|  | Experimental |  | 9.5 (1.00) | 6.5 (1.00) | -2.961 | 0.003** |

*p-value is significant at p<0.05

*p-value is significant at p<0.01

**DISCUSSION**

The spinal change pattern results were investigated in this study using TruPosture Smart Shirt and mobile apps. The reason for choosing this method is that the spinal sitting pattern is limited to laboratory and clinical settings only [2,13]. According to Ma'arof et al. [12], the research field provides vital outcomes and real-world motorcycling information. Thus, to fill the research gap, TruPosture Smart Shirt is the most suitable method to monitor the spinal change pattern among motorcyclists on-road with the application of real working conditions.

The present study found that the existing seat contributes to the huge changes in Sensor 1 (Thoracic 1) throughout 20 minutes of riding for both groups. This could be explained when the riders applied a slumped posture at the end of the 20 minutes of riding due to muscle fatigue while adopting an upright posture without back support. Kwon et al. [14] explained that the thoracic angle is usually related to the outcome of a slumped posture. These findings were supported by Ma'arof et al. [15], who reported that an upright riding posture involved higher muscular activity compared to the forward-lean posture, which makes riders muscle less comfortable, leading to fatigue. Shoulder stiffness is frequently encountered by motorcyclists, including young and healthy riders [16]. Therefore, officers using existing seat proved that they could not maintain an ideal and good posture while riding a motorcycle, indicating the leading cause of concern for this problem.

The application of lumbar support with a built-in massager system showed a lower deviation angle in the trend compared to the group without intervention. This happened because the upright posture without back support (exiting seat) leads to the rider's unnatural spinal curve. This can be explained by the fact that this prototype helps correct the spinal posture (upright) of motorcyclists better than the absence of lumbar support. These findings are parallel with the previous research that reported the lumbar support on a motorcycle seat was capable of providing good posture and reduce muscle discomfort of riders during the riding process [6]. This is supported by Ceunen et al., (2014), who stated that the upright sitting posture with lumbar support is efficient to change pelvis and spinal structure to their natural curve during sitting, which reduces the load on the ischial tuberosity and lower spinal, reduce muscular activity, maintained lumbar lordosis; thus, potentially reduce the risk of developing low back pain. Alyami and Albarrati [16] also found that the workstation would be safer when the posture's biomechanical risk could be reduced by applying ergonomic rules in design that would support healthy (ideal) body posture.

A statistically significant difference was noted throughout this research in all sensors at the 0^th^, 10^th^ and 20^th^ minutes (p<0.05) between pre-test and post-test measurement in the experimental group. This proved that the intervention of the prototype seat in the current study could effectively maintain spinal posture in an upright riding position throughout 20 minutes riding session. The value of spinal angle deviation between the pre-test and post-test in the experimental group throughout 20 minutes of riding also showed that the slumped spinal posture could be prevented with lumbar support and a built-in massager system, as shown in Figure 5.

**Figure 5. Posture after 20 minutes of the riding session**

(a) Existing seat
(b) Prototype seat (lumbar support with build-in massager system)

Another crucial finding in this study involved applying the massager system (4 minutes off to 1 minute on), whereby the results of the experimental post-test group showed relatively not much deviation compared to the other groups. The graph showed that the reading of the deviation of spinal angle was reduced after every four minutes. This could be possibly due to the massager in the lumbar system improves spinal posture from an awkward posture (slump) over time. These results proved the theory that the massager in the lumbar area could reduce muscle fatigue, increase blood flow and oxygenation, and maintain body posture effectively [6-8].

Contrarily, Tanaka et al. [19] reported differences that suggested the lumbar massage failed to exert significant changes in any electromyographic (EMG) measurement. Besides that, Van Poppel et al. [20] reported that lumbar support was not useful in preventing low back pain. The outcome of this study contradicted the study by Kolich et al. [21], where they pointed out that one minute of lumbar massage in every five minutes of the driving session has a beneficial effect on the lower back muscle activity. A study conducted by Franz et al. [22] suggested that lumbar support usage with lightweight massager in automobile seats has been reported to decrease muscle discomfort during the driving process and improve lumbar lordosis.

**CONCLUSION**

In conclusion, there was a significant difference in the spinal angle profiles between the pre-test and post-test sessions in the experimental group. Thus, this proved that a prototype intervention (lumbar support with built-in massager system) successfully maintains and improves the spinal posture angle ergonomically throughout 20 minutes of riding. Although there is a positive outcome in this research, the evidence of the effectiveness of lumbar support with a built-in massager system to reduce muscle fatigue, pressure muscle activity is still scarce. Hence, further study in the artificial laboratory and clinical settings as well as virtual testing is needed to support these findings.

**ACKNOWLEDGEMENTS**

The author would like to thank all of the people who were involved in this study especially the Royal Malaysian Police (RMP).

**REFERENCES**

[1] Fernandes RD, da Silva Pataro SM, De Carvalho RB, Burdorf A. The concurrence of musculoskeletal pain and associated work-related factors: a cross-sectional study. BMC Public Health. 2016 Dec;16(1):1-9.
[2] De Carvalho DE, Callaghan JP. Spine posture and discomfort during prolonged simulated driving with self-selected lumbar support prominence. Human factors. 2015 Sep;57(6):976-87.
[3] Guo LX, Dong RC, Zhang M. Effect of lumbar support on seating comfort predicted by a whole human body-seat model. International Journal of Industrial Ergonomics. 2016 May 1;53:319-27.
[4] Sprigle S, Sonenblum SE, Feng C. Pressure redistributing in-seat movement activities by persons with spinal cord injury over multiple epochs. PloS one. 2019 Feb 13;14(2):e0210978.
[5] Wang SJ, Sommer B, Cheng W, Schreiber F. The Virtual-Spine Platform—Acquiring, visualizing, and analyzing individual sitting behaviour. PloS one. 2018 Jun 13;13(6):e0195670.
[6] Karuppiah K, Salit MS, Ismail MY, Ismail N, Tamrin S. Evaluation of motorcyclist's discomfort during prolonged riding process with and without lumbar support. Anais da Academia Brasileira de Ciências. 2012 Dec;84(4):1169-88.
[7] Donnelly CJ, Callaghan JP, Durkin JL. The effect of an active lumbar system on the seating comfort of officers in police fleet vehicles. International journal of occupational safety and ergonomics. 2009 Jan 1;15(3):295-307.
[8] Durkin JL, Harvey A, Hughson RL, Callaghan JP. The effects of lumbar massage on muscle fatigue, muscle oxygenation, low back discomfort, and driver performance during prolonged driving. Ergonomics. 2006 Jan 15;49(1):28-44.
[9] Mansfield N, Sammonds G, Nguyen L. Driver discomfort in vehicle seats–Effect of changing road conditions and seat foam composition. Applied ergonomics. 2015 Sep 1;50:153-9.
[10] Rashid H, Omar AR, Ahmad IN, Mohamed Z, Wan Fauzi WM, Mahmud Z, Haron R. Motorcyclists’ Prolonged Riding Simulation: The Setup and Procedures. Journal of Mechanical Engineering (JMechE). 2017(5):197-208.
[11] Gouvêa GR, Vieira WD, Paranhos LR, Bernardino ID, Bulgareli JV, Pereira AC. Assessment of the ergonomic risk from the saddle and conventional seats in dentistry: A systematic review and meta-analysis. PloS one. 2018 Dec 17;13(12):e0208900.
[12] Ma’arof MI, Omar AR, Ahmad IN, Jaafar R, Fauzi WM, Rahim AA, Rashid H, Mahmud Z. The motorcycle design parameter database (MDPD) for different motorcycle models. Procedia Manufacturing. 2015 Jan 1;3:2581-8.
[13] Mörl F, Bradl I. Lumbar posture and muscular activity while sitting during office work. Journal of Electromyography and Kinesiology. 2013 Apr 1;23(2):362-8.
[14] Kwon Y, Kim JW, Heo JH, Jeon HM, Choi EB, Eom GM. The effect of sitting posture on the loads at cervicothoracic and lumbosacral joints. Technology and Health Care. 2018 Jan 1;26(S1):409-18.
[15] Ma’arof MI, Omar AR, Haron R, Husain H, Wahab MA. Muscular Activities Measurements of Forward Lean and Upright Sitting Motorcycling Postures via Surface Electromyography (sEMG). InMATEC Web of Conferences 2017 (Vol. 135, p. 00046). EDP Sciences.
[16] Diyana MA, Karmegam K, Shamsul BM, Irniza R, Vivien H, Sivasankar S, Syahira MP, Kulanthayan KC. Risk factors analysis: Work-related musculoskeletal disorders among male traffic policemen using high-powered motorcycles. International Journal of Industrial Ergonomics. 2019 Nov 1;74:102863.
[17] Ceunen E, Zaman J, Vlaeyen JW, Dankaerts W, Van Diest I. Effect of seated trunk posture on eye-blink startle and subjective experience: comparing flexion, neutral upright posture, and extension of the spine. PloS one. 2014 Feb 7;9(2):e88482.
[18] Alyami H, Albarrati AM. Comparison of Spinal Angles in a Typing Task on a Laptop and a Desktop Computer: A Preliminary Study. American Journal of Occupational Therapy. 2016 Nov 1;70(6):7006350020p1-8.
[19] Tanaka TH, Leisman G, Mori H, Nishijo K. The effect of massage on localized lumbar muscle fatigue. BMC complementary and alternative medicine. 2002 Dec;2(1):1-8.
[20] Van Poppel MN, Hooftman WE, Koes BW. An update of a systematic review of controlled clinical trials on the primary prevention of back pain at the workplace. Occupational Medicine. 2004 Aug 1;54(5):345-52.
[21] Kolich M, Taboun SM, Mohamed AI. Low back muscle activity in an automobile seat with a lumbar massage system. International journal of occupational safety and ergonomics. 2000 Jan 1;6(1):113-28.
[22] Franz M, Zenk R, Vink P, Hallbeck S. The effect of a lightweight massage system in a car seat on comfort and electromyogram. Journal of manipulative and physiological therapeutics. 2011 Feb 1;34(2):107-13.
