# Supplementary material for: Effectiveness of lumbar support with built-in massager system on spinal angle profiles among high-powered traffic police motorcycle riders: A randomised controlled trial
Source: PLoS One. 2021 Oct 19;16(10):e0258796. doi: 10.1371/journal.pone.0258796 (PMC8525748; doi:10.1371/journal.pone.0258796)
Supplement: Attachment — Submitted filename: Response to reviewer.docx [file pone.0258796.s003.docx]

Dear Dr Ahmed Mancy Mosa (Academic Editor),

Thank you for giving me the opportunity to submit a revised draft of my manuscript titled ***Assessment of spinal angle profiles among high-powered traffic police motorcycle riders***. We appreciate the time and effort that you and the reviewers have dedicated to providing your valuable feedback on my manuscript. We are grateful to the reviewers for their insightful comments on our paper. We have been able to incorporate changes to reflect most of the suggestions provided by the reviewers. We have highlighted the revisions within the manuscript. The line mention in the author respon

Here is a point-by-point response to the reviewers’ comments and concerns.

| **No.** | **Comments from Reviewer 1** | **Author response** |
| --- | --- | --- |
| 1. | What is the new contribution of this study? | Thank you for pointing this out. The new contribution of this study is:   - The application of the method (Truposture smart shirt) in spinal profile measurement was evaluated in an occupational setting (on-the-road) which riding in a real working condition was applied. - In motorcycling industry, new invention for an ergonomic motorcycle seat had been developed and tested. |
| 2. | What was previously known about the ergonomic function and effect of the prototype seat, and how will this study add to the existing knowledge? Why is it important to measure the spinal angle profile? | We agree with this comment. Therefore, the relevant justification has been made in the introduction section (line 92- 99). |
| 2. | The problem statement highlighted in the introduction does not match the aim of the study which is an experimental study comparing between an intervention and control group. | We agree with this comment and has made changes accordingly in introduction section (line 94-96). |
| 3. | Please clarify whether there is any sample size calculation undertaken prior to the study, and provide the relevant references for the sample size calculation. What was the power of the study based on the sample size calculation? | You have raised an important point here. We agree with this comment and the sample size calculation has been added in sample size section (line 122-129). |
| 4. | Please provide adequate references for the prototype and the TruPosture app. | Adequate references for the prototype and Truposture app have made (line 186-189). |
| 5. | Please provide a detailed explanation on the recruitment process, and the allocation to control and intervention group. | Thank you for pointing this out. The recruitment process has been explained accordingly (line 131-158). |
| 6. | What was the dependent variable in the statistical analysis. please provide the operational definition of the dependent variable. | The dependent variable and the operational definition have been added in the statistical analysis as suggested (line 223-225). |
| 7. | Please provide the general description of your study participants in the results section, and describe the characteristics between the experimental and control group to show that they are comparable. | Thank you for pointing this out. The general description of the participants has been discussed and tabulated in Table 1 (line 234-242). |
| 8. | Do you expect any confounding factors in your final analysis to look at the difference in outcome between the intervention and control group? What are the appropriate statistical tests to be used to control for the confounders, if there is any? | The confounding factors in this study was expected with the difference age, gender, BMI and present of low back pain. However, these criteria were controlled based on inclusion and exclusion criteria during sampling (line 144-121). |
| 9. | please explain clearly the within and between groups comparison in the final analysis of the study outcome. | Thank you for pointing this out. We agree with this comment and has made changes accordingly in results and discussion section (line 316-330). |
| 10. | Please align the discussion with the aim of the manuscript. What is it that you plan to highlight? is it about the usability of the TruPosture smart Shirt in measuring posture, or it is about showing the effect of the prototype seat on posture of the police riders? | Agree. We have, accordingly, revised the discussion part and removed the unrelated part. |
| 11. | What is the study limitation? | Thank you for pointing this out. We have added the study limitation accordingly in the discussion section (line 350-359). |
| 12. | Please revise the manuscript title to reflect on the aim and experimental design. | We agree with this comment. Therefore, we have revised and changed the manuscript title into “Effectiveness of lumbar support with built-in massager system on spinal angle profiles among high-powered traffic police motorcycle riders: A randomised controlled trial” |

| **No.** | **Comments from Reviewer 2** | **Author response** |
| --- | --- | --- |
| 1. | • There is no mention on the specific study design and how the randomisation is done (authors only mentioned randomisation once in Figure 2). Is this a randomised controlled trial? Why is it not mentioned in the title? Has it been registered in any trial’s registry? | Thank you for the input. Yes, it is randomised controlled trial. However, we apologise for overlooking this matter. Thus, the title and study design have been revised as suggested (line 108-112).  It has not been registered in any trial’s registry. We are sorry for this and hope for your understanding. Nevertheless, the sampling method is approved and supported by the Ethical Committee of Universiti Putra Malaysia after consulting their expertise. |
| 2. | • There is no mention of how the sample size is calculated. How is the sample size being determined? Is the sample size adequate to represent the study population? Is the sample size adequate to meet the assumption of statistical analyses and come to conclusion? | You have raised an important point here. We agree with this comment and the sample size calculation has been added (line 122-129). |
| 3. | • Eligibility criteria is not clear (authors only mention no history of MSD and low back pain, but what about baseline age, height, weight, BMI, fat percentage, gender, ethnicity, years of service, and other factors that might influence the spinal angle profile?). | You have raised an important point here. We agree with this comment and has included relevant information and explanation accordingly (line 114-121).  However, due to time (pandemic outbreak) and budget constraints (e.g: fat percentage analyser), we are unable to include all aspects of the problem and other factors. Thank you for pointing this out. For future studies, this will be a great insight and we will be sure to use it. Also, we will highlight this matter as the limitation in this study. |
| 4. | • There is no mention on how the recruitment is done. Is it convenience? Can it be representative of the study population? Who enrolled the participants? | Thank you for pointing this out. The participant recruitment has been included and explained accordingly (131-158). |
| 5. | • How is the allocation to the control and intervention group being done? Is there any blinding involved? What are the methods used to do randomisation and type of randomisation (when allocating participants to control or intervention group)? The authors did not mention who did the randomisation and who assigned the participants interventions. | Thank you for pointing this out. The participant recruitment has been included and explained accordingly (131-158). |
| 6. | There is no mention of the time period of data collection defining recruitment, pre and post-test. How long is the gap between pre and post-test? | Thank you for pointing this out. The participant recruitment has been included and explained accordingly (line 144). |
| 7. | What specific time of the day the experiment being conducted? (is the timing and duration of work on that particular experiment day influence the spinal angle profiles?) What is the justification for 20 minutes duration of the experimental riding session? (authors mention riders spend at least 5 hours per day on motorbikes). | You have raised an important point here. We agree with this comment and have included the relevant information and justification in data collection section (line 205-209). |
| 8. | • The author did not mention anything on the instrument validation and calibration. How are the sensors being placed? Is it done by the same person for both control and intervention groups? Has the apps being tested and validated before? How accurate is the reading? | We agree with this comment. Therefore, we have discussed this information in TruPosture section (line 186-192). |
| 9. | • The author did not mention whether both groups are being assessed by the same person (e.g. posture for riding). If it’s not done by the same person, do the researchers take into account inter-rater reliability? | You have raised an important point here. Thus, the relevant information has been added accordingly (line 209-210). |
| 10. | b) Discussion There is no mention or any discussion on the experiment’s limitations in the discussion part. The authors did not discuss potential source bias and confounder, threat to validity that might compromise the findings. | Thank you for pointing this out. We have added the study limitation accordingly (line 350-364). |
| 11. | c) Results  • The authors did not report the sociodemographic and baseline characteristics of both control and experimental group. How does the researcher ensure that both groups are similar at baseline? How can the researcher then conclude that the outcome is due to their intervention, rather than existing differences? Are there any additional methods of analyses such as subgroup analyses to account for confounder or differences in the baseline? | Thank you for pointing this out. The general description of the participants has been discussed and tabulated in Table 1 (line 234-242). |
| 12. | • There is no justification on why non-parametric statistical analyses was chosen. What are the assumptions and limitation of the analysis? Have all the criteria being met? | We agree with this comment and has made changes accordingly in statistical analysis section (line 218-223). |
| 13. | • Too much data being presented that can be summarised in a sentence or in simpler table. For example, Table 1 presents a range of minimum and maximum value for each reading throughout 20 minutes – can be summarised by providing the mean/median value. Easier to interpret and understand, rather than the reader have to go thru each range. | Agree. We have removed this table accordingly and the mean value of spinal posture angle changes throughout 20 minutes riding for both groups had already presented in Figure 3 (line 250). |
| 15. | d) Others  • Figure 1 – no legend to help reader understand the figure. Which line belongs to control and intervention group?  • Figure 2 – suggest authors to follow CONSORT flow diagram format (more details) | Actually Figure 1 is an example of Truposture mobile apps interface and not related with the control and intervention group results.  Thank you for your suggestion. Thus, Figure 2 has been changed and followed CONSORT format (Fig1: Line 146). |

| **No.** | **Comments from Reviewer 3** | **Author response** |
| --- | --- | --- |
| 1. | Abstract Line 30: the purpose of the study described in the abstract does not match with what was done in the methods. This was an intervention study and I suggest reformatting the purpose of the study. | The purpose of the study has been reformulated accordingly (line 30-31). |
| 2. | Line 40: Are we really interested in a pre-test VS post-test analysis (within group analysis)? Reporting the between group comparison is more insightful… | Thank you for pointing this out. Thus, results and discussion related to comparison between groups have been added accordingly |
| 3. | Introduction  Provide some statistics of MSD/low back in the population | The statistics of MSD and low back pain among traffic police riders have been provided as suggested. (line 78-85) |
| 4. | Line 51: provide a full meaning of MSD | The full meaning of MSD has been provided as suggested (line 50). |
| 5. | Line 59: I believe it was meant to be “been” instead of “seen” | Thank you for the comment. The relevant word has been changed accordingly (line 104-106). |
| 6. | Line 61: has instead of have | Thank you for the comment. The relevant word has been changed accordingly. |
| 7. | Line 67: delete “the” | Thank you for the comment. The relevant word has been deleted accordingly. |
| 8. | Line 81: The purpose of the study needs to be reformulated | The purpose of the study has been reformulated accordingly. |
| 9. | The introduction lacks content. The consequences of increased lumbar lordosis are not fully described. There are some grammatical errors that need to be corrected. | Thank you for pointing this out. The consequence of decreased lumbar lordosis has been explained accordingly (line 52-60). |
| 10. | Materials and Methods  More information on the eligibility criteria (inclusion and exclusion criteria) is needed | You have raised an important point here. We agree with this comment and has included relevant information and explanation accordingly (114-121). |
| 10. | Any reference for the TruePosture mobile app? Has this app been used before in any study? What are the validity and reliability properties of the app? | Adequate references for the Truposture app have been added and discussed (line 186-189). |
| 11. | How were the groups defined? Were the participants randomly assigned to the groups? What are the baseline characteristics of the control and experimental groups? | We agree with this comment. Thus, the relevant information has been added and explained accordingly. |
| 12. | Why did the authors not perform a between-group analysis? That comparison is more interesting than all those pre-test vs post-tests performed. | Thank you for pointing this out. Thus, results and discussion related to the comparison between groups have been added accordingly. |
| 13 | Results  The data reported does not indicate whether the spinal change pattern in the intervention group is superior to the control group. | Thank you for pointing this out. Thus, results and discussion related to the spinal change pattern between groups have been added accordingly. |
| 14. | Discussion and conclusion  Not sustained by the analysis and results presented | The analysis and results presented have been revised. |

| **No.** | **Comments from Reviewer 4** | **Author response** |
| --- | --- | --- |
| 1. | Context and research gap were not indicated. | The research gap was explained in line 85. |
| 2. | Type of study, sampling method and data collection method were not specified.  It has major methodological defect.  Type of study, sampling method and data collection method were not specified  Gold standard of experimental study was not succinctly stated. | Thank you for pointing this out. We agree with this and have incorporated your suggestion throughout the manuscript accordingly. |
| 3. | Major statistical analysis was not indicated. Why Wilcoxon signed rank test and median was used? | The reason the analysis was chosen has been discussed in details (line 218-223). |

| **No.** | **Comments from Reviewer 5** | **Author response** |
| --- | --- | --- |
| 1. | Reviewer #5: Line 51: Please indicate the full meaning of MSD when been used for the first time in write up. | Thank you for pointing this out. The change has been made accordingly (line 50). |
| 2. | Line 178: Consider replacing "respondents" with "subjects" which best suits the study and its concept | Thank you for pointing this out. The change has been made throughout the manuscript accordingly. |
| 3. | Please give reasons for the sample size choice and indicate the exclusion and inclusion criteria for participating in the study | You have raised an important point here. We agree with this comment and has included relevant information and explanation accordingly (line 114-129). |
| 4. | Line 243: "The value of spinal angle deviation between the pre-test and post-test ....." Indicate the angle of deviation to make your point clear | Thank you for the comment. Actually, we want to highlight the effect in the experimental group. However, we have made some changes based on your suggestion  *“The value of spinal angle deviation in the experimental group….”* |
| 5. | There are some typographical errors indicated in the attached document. Please revise them accordingly | Thank you for pointing this out. The typological error has been revised accordingly. |
