# Supplementary material for: Effectiveness of lumbar support with built-in massager system on spinal angle profiles among high-powered traffic police motorcycle riders: A randomised controlled trial
Source: PLoS One. 2021 Oct 19;16(10):e0258796. doi: 10.1371/journal.pone.0258796 (PMC8525748; doi:10.1371/journal.pone.0258796)
Supplement: Attachment — Submitted filename: Manuscript Revision 1_Edits and Comments.docx [file pone.0258796.s004.docx]

**Effectiveness of lumbar support with built-in massager system on spinal angle profiles among high-powered traffic police motorcycle riders: A randomised controlled trial**

Nur Athirah Diyana Mohammad Yusof^1¶^, Karmegam Karupiah*^1¶^, Shamsul Bahri Mohd Tamrin ^1&^, Irniza Rasdi^1^, Vivien How^1&^, Sivasankar Sambasivam^1&^, Putri Anis Syahira^1¶^, Kulanthayan K. C. Mani^2&^, Hassan Sadeghi Naeini^3&^, Dayana Hazwani Mohd Suadi Nata^4&^

^1^Department of Environmental and Occupational Health, Faculty of Medicine and Health Sciences, Universiti Putra Malaysia, UPM Serdang, Selangor, Malaysia

^2^Department of Community Health, Faculty of Medicine and Health Sciences, Universiti Putra Malaysia, UPM Serdang, Selangor, Malaysia

^3^Industrial Design Department, School of Architecture & Environmental Design, Iran University of Science & Technology, Tehran, Iran

^4^Faculty of Industrial Science and Technology, Universiti Malaysia Pahang, Lebuhraya Tun Razak, Gambang Kuantan Pahang, Malaysia

*Corresponding author

Email: [megam@upm.edu.my](mailto:megam@upm.edu.my)

^¶^These authors contributed equally to this work.

^&^These authors also contributed equally to this work.

**ABSTRACT**

The study aimed to evaluate the effect of lumbar support with a built-in massager system on spinal angle profiles among traffic police riders. A randomised controlled trial (pre-testpost-test) was used to assess spinal angle pattern while riding the high-powered motorcycle for 20 minutes. Twenty-four traffic police riders were randomly selected to participate and 12 riders were assigned to the control group and 12 riders to the experimental group. The pre-test and post-test were conducted at a one-week interval. Each participant was required to wear a TruPosture Smart Shirt (to monitor spinal posture). The TruPosture Apps recorded the spinal angle pattern. The data indicated that the police riders using motorcycle seat with lumbar support and built-in massager system showed a huge improvement in maintaining posture which only involves slight spinal angle deviation changes from the spinal reference angle throughout the 20 minutes ride. There were significant differences in all sensors between the control group and experimental groups (p<0.05) and within the experimental group. According to the findings, it can be said that the ergonomic intervention prototype (lumbar support with built-in massager system) successfully helps to maintain and improve the natural curve of the spinal posture. This indirectly would reduce the risk of developing musculoskeletal disorders and spinal injury among traffic police riders.

Keywords: posture, lumbar support, massage system, intervention, seat

**Introduction**

The riding posture is very important because an awkward riding posture may cause discomfort to the riders and increase the risk of developing musculoskeletal disorders (MSD) in long-term exposure. De Cássia et al. [1] stated that body discomfort is an indicator of many MSD due to prolonged discomfort. A sustained flexion of lumbar lordosis (low back) during driving usually is the main factor in the generation of low back pain [2]. According to Makhsous et al. [3], sitting decreases lumbar lordosis compared to the standing posture, resulting in increased disc pressure and low back muscle activity. This is because while sitting, the ischial tuberosity mainly supports the majority of the upper body weight. Increased pressure in this area is significantly associated with increased spinal load [4]. Riding a motorcycle exposes the officers to excessive physical demand, especially for prolonged periods. They will tend to feel discomfort and possibly fatigue while sitting in the same position with restricted movements. Thus, to improve spinal posture and comfort in transportation settings, seat design has a significant role and efficiency.

A well-designed seat would decrease pressure within the spinal discs, spinal ligaments, and gluteal muscle [5-8]. Lumbar support is the solution for the problem, whereby the weight and pressure of the trunk are taken by the back support and increased lumbar lordosis. The use of a vehicle seat (lumbar support) has been significantly associated with the reduction of muscle discomfort and low back pain during prolonged riding and driving journey [5,8]. Adjustable lumbar support with extra cushioning, which provided a massage-like comfort, has been proved to reduce transmission of vibration, which indirectly increases muscle comfort by improving oxygen and blood flow to the tissue [9,10]. According to Mansfield et al. [11], the seat shape, user suitability, seat material, duration of sitting in the same position, vibration, and posture changes are among the factors that influence seat comfort.

Traffic police is a police officer who serves to enforce the rules related to traffic. They have to perform various work tasks, such as escorting important person to any events, patrol selected locations, and finding any road offenders. All this work task requires them to use a motorcycle as their vehicle. At present, the officer riding a motorcycle takes at least five hours per day during duties. Therefore, traffic police riders have to endure prolonged riding duration. Rashid et al. [12] believed that continuous riding of a standard motorcycle for an extended time would result in a high level of postural fatigue and health problems. From a previous research study on the CBX 750P21 motorcycle, it was found that 88.3% of traffic police riders suffered from MSD with 34.3% of them suffering from lower back pain due to static posture and prolonged sitting while riding the motorcycle [13]. Another previous study also revealed that more than half (54.7%) of the traffic police riders rode high-powered motorcycles for an average of 5.64 hours per day with a fixed posture leading to increased discomfort from prolonged sitting that enhanced muscle fatigue [14]. This showed that most of their working time involves riding motorcycles. However, to the best of the authors' knowledge, no research has been done on the spinal riding posture during a motorcycle ride, especially among police officers in Malaysia who use a motorcycle to perform their duties daily.

To solve this problem, ergonomics is the best solution for scientific research in man-machine interaction at the workplace since this field involves fitting machine to workers comfortability to improve their working performance, reduce fatigue, and stress [15]. The ergonomic application is very significant in areas involving prolonged and static riding activities that directly affect the riders' healthy spinal posture and reduce muscle fatigue. Lumbar support with a built-in massager system is an intervention in this study to solve the ergonomic issue among motorcycle riders. The use of a back (lumbar) support has been proved to help maintain the natural spine lordotic lumbar curvature of the person while sitting compared to those without back support [16]. In a clinical and laboratory setting, automobile seat massage is utilised widely, and it has been proved efficacious in recovering from postural fatigue [9,10]. However, research and data on the effectiveness of this intervention seat, lumbar support with a built-in massager system, in an in-field setting (on-the-road) are insufficient. Thus, the present study integrates a massager system and lumbar support with the existing seat which an on-road test was conducted to maintain and support the spinal posture of the riders more efficiently as the test involved prolonged and static riding. In this study, the spinal angle profile was measured and assessed to determine the effectiveness of the prototype seat in supporting the spinal body posture of the riders throughout 20 minutes motorcycle ride. Thus, the present study seeks to evaluate the effect of lumbar support with a built-in massager system (prototype seat) among traffic police riders.

**Materials and methods**

**Study Design**

A randomised controlled trial was conducted among 24 traffic police riders who ride a high-powered motorcycle (Honda CBX 750). The subjects were randomly assigned to the control group (12 riders) and experimental group (12 groups). Data collection commenced in March 2020 and finished in July 2020.

**Eligibility Criteria**

Eligible subjects were male riders only because almost 90% of traffic police riders are male. BMI was between 18.5 to 29.9 kg/m^2^. The age group recruitment was between 20 and 39 years old because more than 40 years old are usually prone to experiencing low back pain due to the age factor and changes in spinal posture [17]. At least one year of experience in high-powered motorcycle riding. Exclusion criteria were the presence of any injury (under treatment or taking any medication related to muscle pain), especially lower back pain, in the 12 months preceding this survey.

**Sample size**

To calculate the appropriate sample size, we use the formula of group comparison guidelines in a study done by Donnelly et al. [9] as a reference. The estimated SD (30.2), estimated larger mean (57.2) and estimated lower mean (19.0) were used in this formula. In this study, the desired power is 80% and the significant level is 95%. Thus, each experimental and control group would have 10 subjects. However, an additional 20% dropout rate was added in the sample size calculation. Thus, each experimental and control group will have 12 subjects. Therefore, a total of 24 subjects was selected in this study.

**Participant Recruitment**

The recruitment strategy used involvedtaking the name list of all officers working at the Kuala Lumpur Traffic Police Station. The name lists were obtained from the Human Resources Department. However, officers from the escort department only were chosen to ensure the consistency of data in which different department have different task or job and the duration of riding police’s motorcycle. Only respondent who fulfilled the criteria were selected for the study. For qualification criteria, respondents were asked to fill up the pre-survey form and the BMI were measured by the main researcher. After the eligible subjects were selected, then a main researcher was randomly assigned them into control or experimental group. This study's sampling method was simple random sampling, in which all the subjects' names were numbered into pieces of paper and put in a container. Then, the subjects were chosen randomly into the experimental or control group by Fishbowl Technique. Each subject in the experimental group received lumbar support with a built-in massager system during post-test. The pre-test and post-test were conducted at a one-week interval. Detail about study flow was illustrated in the CONSORT diagram, Fig 1.

**Fig 1. CONSORT diagram**

Subjects (n=12) allocated into the control group had undergone post-test session without the lumbar support with a built-in massager system (existing seat) during 20 minutes riding session. On the other hand, the experimental group (n=12) had undergone a post-test session with the lumbar support with a built-in massager system (prototype seat) for 20 minutes riding session.

Before the experimental session began, subjects were allowed to choose the Truposture smart shirt that fits their body. This was done to ensure the point of each sensor would be placed in the correct position of the spine. There were no cases of unable to follow up or incomplete experimental testing as planned in this study. All subjects completed the follow-up stage (n=24) in both intervention groups. This study had no excluded analysis cases as all the data were successfully recorded without any missing data.

**A prototype**

The lumbar support on the prototype seat can be adjusted either upward and downward according to the lumbar height position for the comfortability of the riders [8]. There is also a massage fitted inside the lumbar support with two rotating balls [10]. The lumbar massage was set at a ratio of 1 minute on and 4 minutes off [18]. It was used for 20 minutes in the experimental riding session [19]. It can support up to 150 kg body weight.

**TruPosture**

A spinal posture angle was obtained using the TruPosture Smart Shirt connected with the TruPosture mobile app among the police riders while riding a motorcycle with an existing seat for both groups; control and experimental during the pre-test. Then, during the post-test, the control group used the same motorcycle seat, and the experimental group used a prototype seat containing adjustable lumbar support with a build-in massager system. The TruPosture Smart Shirt covered different regions of the spinal alignment, with five nano-sensor technology attached undershirt. The five sensors cover the thoracic vertebrae, lumbar, and the pelvic regions (T1, T8, L1, L3, and Pelvis).

The technology helped the researcher monitor the entire spinal’s alignment and curvature in several modes, including standing and sitting. The TruPosture mobile apps interface (Fig 2) and Windows software were utilised as these tools help the researcher track the posture and record the movement of posture in real-time. The sensors detect the real-time changes of spinal posture angle based on the spinal movement.

**Fig 2.** **TruPosture Mobile Apps Interface**.

Based on Fig 2, the blue curve represents the spinal reference posture (ideal posture) at 0 minutes, and the orange curve indicates the actual spinal riding posture throughout 20 minutes. The number on the left and right side of the interface represents the angle reading for each spinal point sensor. The positive and negative values of angle indicate the spinal position, which is forward and backward respectively.

This equipment had been tested the validity and approved by the previous study which was suitable for monitoring the posture of the spine [20]. The reason for choosing this method is that the spinal sitting pattern studies were limited to laboratory and clinical settings only [2,21]. According to Ma'arof et al. [22], the research field provides vital outcomes and real-world motorcycling information. Thus, TruPosture Smart Shirt is the most suitable method to monitor the spinal change pattern among motorcyclists on-road with the application of real working conditions.

**Data Collection**

In this study, the spinal reference posture at the 0 minutes riding session was set first. The subjects wore the TruPosture Smart Shirt during the monitoring, according to the ideal riding posture. An upright riding posture is the best posture in riding this type of motorcycle based on the standard operating procedure (SOP) of Honda CBX750P motorcycle riding. According to Ma’arof et al. [22], a good riding posture for the upright posture is that the arms should bend slightly when gripping the throttle and sitting in the upright position. The shoulders and elbows are held easily on the holds without exceeding or over-expanding the elbows. Elbows are flexed, and lower arms are parallel to the ground. Furthermore, the legs are held near the fuel tank while relaxing the hip and pelvis. This posture was set as a spinal reference posture for riding, and the angle for each sensor was recorded. However, this is the real problem for the riders, as they could not maintain the ideal posture in medium- and long-distance trips. Thus, the subjects were asked to ride a motorcycle for only 20 minutes. This is because the average commute time of traffic police riders for one task is less than 30 minutes. Moreover, According to Deros et al. [19], 82.5% of the discomfort variance divergence is accounted for after being seated for 20 minutes and it takes less than five minutes in postural changes without back support. The riding posture was assessed by the same person (an ergonomist) in the pretest-posttest session.

This study was conducted at the MEX highway route (the route between the commercial centre of Kuala Lumpur to the Federal Administrative capital of Putrajaya and Kuala Lumpur International Airport), with good road condition and maintenance. The route was chosen as it was commonly used in their work task as escort riders. The pre-post test results were then compared between the two groups.

**Statistical Analysis**

The collected data from the questionnaire and measurement were analysed using the IBM SPSS Software (Version 26). The Shapiro-wilk test was used in this study to determine the normality of data distribution for variables. The normality of data distribution was assumed when the p-value was more than 0.05. The present study found that the data variables were not normally distributed. Thus, a non-parametric test was used in this study. The data collected were analysed using the Mann-Whitney test and Wilcoxon signed-ranked test to verify a statistically significant difference between and within the control and experimental groups. The dependent variable of this study was the spinal angle (0^th^ and 20^th^ minutes). The measurement of the spinal angle was done by using Truposture smart shirt. The study was conducted using a 95% confidence level, 80% of power, and the results of p≤0.05 were considered significant.

**Ethics Approval**

This study was submitted and approved by the Ethics Committee, Universiti Putra Malaysia (reference number: UPM/TNCPI/RMC/JKEUPM/1.4.18.2 (JKEUPM)). Permission from the subjects of this study was obtained with their written consents before the study was conducted. Privacy of the information and confidentiality of the subjects were and are always protected.

**RESULTS**

**Subjects**

In total, 24 traffic police riders were included in the study, and there were no drops out. All subjects enrolled completed the study protocol as planned. The subjects’ demographic data from both the control group and the experimental group are tabulated in Table 1; no significant differences were observed between the two groups.

**Table 1. Distribution of socio-demographic and occupational profiles between the control group and experimental group (N=24)**

| **Variable** | **Control group**  **(n=12)** | **Experimental group (n=12)** |
| --- | --- | --- |
| Age (years)  Mean±SD  Range | 30.25±5.72  22-38 | 28.92±3.78  24-37 |
| BMI (kg/m^2^)  Mean±SD  Range | 24.29±2.26  20.55-29.05 | 24.04±3.17  19.38-29.1 |
| Working hours per day (hours)  Mean±SD  Range | 9.17±1.75  8.0-14.0 | 9.50±1.88  8.0-13.0 |
| Riding in a day (hours)  Mean±SD  Range | 4.50±1.64  2.0-8.0 | 5.08±1.97  3.0-8.0 |
| Year of service | 5.67±3.28 | 5.84±2.98 |

**Distribution of five different sensors for spinal posture angle**

The mean spinal posture angle in each sensor for the control and experimental groups is presented in Fig 3. The graph showed that the experimental group (post-test) values are successfully maintained with minimal changes to the spinal posture angle compared to the other groups. The positive and negative values of angle indicate the position of the spinal, which is forward and backwards, respectively.

**Fig 3. Mean spinal posture angle changes throughout 20 minutes riding for pre-test and post-test study.** C_P_0_: a control pre-test group; C_P_1_: a control post-test; E­_P_0_: Experimental pre-test; E_P_1_: Experimental post test

Meanwhile, the line graph in Fig 4 (a–e) show the trend comparison of spinal posture angle deviation throughout the 20 minutes riding session. The line graph compares the level of spinal angle deviation in four groups (control pre-test, experimental pre-test, control post-test, and experimental post-test). Based on the graph line comparison, a deviation of spinal angle increased in each sensor, and Sensor 1 experienced the greatest deviation from the other sensors. From the graph, a deviation of spinal angle on each group shows an upward trend. However, the spinal angle deviation in the experimental (post-test) group is always at a lower level than the other groups. It showed that the experimental group intervention has a huge improvement in maintaining posture, which only involves slight spinal angle deviation changes from the spinal reference angle throughout the 20 minutes ride. The details of the spinal posture angle profile throughout 20 minutes are available as supporting information; S1.

**Fig 4. (a–e). Deviation of spinal posture angle throughout 20 minutes (N=24)**

**Comparison of Five Different Sensors**

Table 2 compares the spinal angle (0^th^ and 20^th^ minutes) between pre-test and post-test study for five different sensors within two groups. As depicted in this table, a Wilcoxon signed-rank test revealed a statistically significant difference between pre-test and post-test session in all sensors at the 0^th^ and 20^th^ minutes in the experimental group (p<0.05). Meanwhile, no statistically significant difference is observed among the control group in all five sensors (p>0.05). The results obtained from a Mann-Whitney test showed there were statistically significant differences in the spinal angle between control and experimental groups for all sensor (p<0.05) throughout 20 minutes of a motorcycle ride. However, no statistically significant difference in spinal angle between the control group and experimental group in the pre-test session (Table 3).

**Table 2. A median angle of five different sensors between pre-test and post-test studies within two groups**

| **Sensor** | **Minute** | **Control** | | | | **Experimental** | | | |
| --- | --- | --- | --- | --- | --- | --- | --- | --- | --- |
|  |  | **Median (IQR)** | | **Z-statistic** | **p-value** | **Median (IQR)** | | **Z-statistic** | **p-value** |
|  |  | **Pre-** | **Post-** |  |  | **Pre-** | **Post-** |  |  |
| 1 | 0 | 25.5 (2.54) | 26.4 (2.57) | -1.193 | 0.471 | 25.7 (2.75) | 20.5 (3.75) | -3.065 | 0.002** |
|  | 20 | 38.5 (2.73) | 38.6 (1.76) | -1.021 | 0.307 | 40.0 (1.75) | 24.5 (1.00) | -3.070 | 0.002** |
| 2 | 0 | 4.0 (2.50) | 4.71 (2.00) | -1.615 | 0.160 | 4.8 (1.75) | 3.71 (1.00) | -2.914 | 0.004** |
|  | 20 | 12.5 (2.70) | 12.0 (3.00) | -1.725 | 0.084 | 12.6 (0.32) | 6.23 (0.98) | -3.061 | 0.002** |
| 3 | 0 | -12.0 (2.75) | -12.0 (3.00) | -0.751 | 0.453 | -13.0 (2.69) | -11.0 (1.75) | -2.754 | 0.006** |
|  | 20 | -3.0 (1.00) | -3.5 (1.00) | -1.308 | 0.191 | -4.00 (1.75) | -10.5 (1.00) | -3.077 | 0.002** |
| 4 | 0 | -16.0 (2.50) | -16.0 (1.75) | -0.206 | 0.837 | -16.0 (2.00) | -15.0 (1.50) | -2.263 | 0.024* |
|  | 20 | -4.0 (2.00) | -4.5 (2.75) | -1.083 | 0.279 | -5.0 (1.75) | -12.0 (2.00) | -3.074 | 0.002* |
| 5 | 0 | 7.0 (1.75) | 8.0 (2.75) | -1.533 | 0.125 | 8.0 (1.75) | 6.0 (1.75) | -2.090 | 0.037* |
|  | 20 | 9.0 (1.00) | 10.0 (2.0) | -1.408 | 0.159 | 9.5 (1.00) | 6.5 (1.00) | -2.961 | 0.003** |

*p-value is significant at p<0.05

**p-value is significant at p<0.01

**Table 3. The comparison of the median spinal angle between control and experimental groups**

| **Sensor** | **Minute** | **Pre-test** | | | | **Post-test** | | | |
| --- | --- | --- | --- | --- | --- | --- | --- | --- | --- |
|  |  | **Median (IQR)** | | **Z-statistic** | **p-value** | **Median (IQR)** | | **Z-statistic** | **p-value** |
|  |  | **Control** | **Experimental** |  |  | **Control** | **Experimental** |  |  |
| 1 | 0 | 25.5 (2.54) | 25.7 (2.75) | -1.172 | 0.241 | 26.4 (2.57) | 20.5 (3.75) | -4.129 | 0.004** |
|  | 20 | 38.5 (2.73) | 40.0 (1.75) | -1.995 | 0.053 | 38.6 (1.76) | 24.5 (1.00) | -4.225 | 0.001** |
| 2 | 0 | 4.0 (2.50) | 4.8 (1.75) | -1.505 | 0.143 | 4.71 (2.00) | 3.71 (1.00) | -2.209 | 0.027* |
|  | 20 | 12.5 (2.70) | 12.6 (0.32) | -1.879 | 0.064 | 12.0 (3.00) | 6.23 (0.98) | -4.197 | 0.002** |
| 3 | 0 | -12.0 (2.75) | -13.0 (2.69) | -1.318 | 0.187 | -12.0 (3.00) | -11.0 (1.75) | -2.385 | 0.016* |
|  | 20 | -3.0 (1.00) | -4.00 (1.75) | -1.792 | 0.079 | -3.5 (1.00) | -10.5 (1.00) | -4.227 | 0.001** |
| 4 | 0 | -16.0 (2.50) | -16.0 (2.00) | -0.412 | 0.681 | -16.0 (1.75) | -15.0 (1.50) | -2.923 | 0.003** |
|  | 20 | -4.0 (2.00) | -5.0 (1.75) | -1.869 | 0.073 | -4.5 (2.75) | -12.0 (2.00) | -4.189 | 0.001** |
| 5 | 0 | 7.0 (1.75) | 7.0 (1.75) | -1.285 | 0.199 | 8.0 (2.75) | 6.0 (1.75) | -2.359 | 0.018* |
|  | 20 | 9.0 (1.00) | 9.5 (1.00) | -1.246 | 0.810 | 10.0 (2.0) | 6.5 (1.00) | -4.078 | 0.001** |

*p-value is significant at p<0.05

**p-value is significant at p<0.01

**Discussion**

The present study found that the existing seat contributes to the huge changes in Sensor 1 (Thoracic 1) throughout 20 minutes of riding for both groups. This could be explained when the riders applied a slumped posture at the end of the 20 minutes of riding due to muscle fatigue while adopting an upright posture without back support. Kwon et al. [23] explained that the thoracic angle is usually related to the outcome of a slumped posture. These findings were supported by Ma'arof et al. [24], who reported that an upright riding posture involved higher muscular activity compared to the forward-lean posture, which makes riders muscle less comfortable, leading to fatigue. Shoulder stiffness is frequently encountered by motorcyclists, including young and healthy riders [13]. Therefore, officers using existing seat proved that they could not maintain an ideal and good posture while riding a motorcycle, indicating the leading cause of concern for this problem.

The application of lumbar support with a built-in massager system showed a lower deviation angle in the trend compared to the group without intervention. This happened because the upright posture without back support (exiting seat) leads to the rider's unnatural spinal curve. This can be explained by the fact that this prototype helps correct the spinal posture (upright) of motorcyclists better than the absence of lumbar support. These findings are parallel with the previous research that reported the lumbar support on a motorcycle seat was capable of providing good posture and reduce muscle discomfort of riders during the riding process [8]. This is supported by Ceunen et al. [25], who stated that the upright sitting posture with lumbar support is efficient to change pelvis and spinal structure to their natural curve during sitting, which reduces the load on the ischial tuberosity and lower spinal, reduce muscular activity, maintained lumbar lordosis; thus, potentially reduce the risk of developing low back pain. Alyami and Albarrati [26] also found that the workstation would be safer when the posture's biomechanical risk could be reduced by applying ergonomic rules in design that would support healthy (ideal) body posture.

A statistically significant difference was noted throughout this research in all sensors at the 0^th^, and 20^th^ minutes (p<0.05) between pre-test and post-test measurement within the experimental group. However, there was no statistically significant difference in spinal angle within the control group. This proved that the intervention of the prototype seat in the current study could effectively maintain spinal posture in an upright riding position throughout 20 minutes riding session. The value of spinal angle deviation between the pre-test and post-test in the experimental group throughout 20 minutes of riding also showed that the slumped spinal posture could be prevented with lumbar support and a built-in massager system.

The analyses between-group differences also found that there was a significant difference in all spinal angle between control and experimental groups throughout 20 minutes of a motorcycle ride. The findings in the current study proved that the prototype seat provides a positive effect on the spinal posture of motorcyclists in adjusted and maintaining their spinal posture which can serve as a channel for the distribution of the force as well as static loading by the body. In this case, the intradiscal pressure could be reduced and the supporting back muscle could be enhanced [27] as shown in Figure 5.

**Figure 5. Posture after 20 minutes of the riding session**

(a) Existing seat
(b) Prototype seat (lumbar support with build-in massager system)

Another crucial finding in this study involved applying the massager system (4 minutes off to 1 minute on), whereby the results of the experimental group showed relatively not much deviation compared to the other groups. The value of spinal angle deviation in the experimental group was reduced after every four minutes. This could be possibly due to the massager in the lumbar system improves spinal posture from an awkward posture (slump) over time. These results proved the theory that the massager in the lumbar area could reduce muscle fatigue, increase blood flow and oxygenation, and maintain body posture effectively [8-10].

Contrarily, Tanaka et al. [28] reported differences that suggested the lumbar massage failed to exert significant changes in any electromyographic (EMG) measurement. Besides that, Van Poppel et al. [29] reported that lumbar support was not useful in preventing low back pain. The outcome of this study contradicted the study by Kolich et al. [18], where they pointed out that one minute of lumbar massage in every five minutes of the driving session has a beneficial effect on the lower back muscle activity. A study conducted by Franz et al. [30] suggested that lumbar support usage with lightweight massager in automobile seats has been reported to decrease muscle discomfort during the driving process and improve lumbar lordosis.

There were several limitations in the present study. Only male traffic police riders were recruited. Thus, it cannot be used to generalise the whole population. A future study might include female traffic police riders and other occupations such as food delivery. Even though we found a positive outcome of this prototype in maintaining spinal posture, since this study was conducted in an on-the-road setting, there was a limited objective measurement that can be done such as the measurement of muscle fatigue using EMG electrode (clinical testing) due to environmental factors such as road condition and vibration. This study was unable to blind the respondents to the condition that they were exposed to, which might impact the posture riding in this study. This is because traffic police riders were aware of which seat they were using, and as such, there was no way to prevent a bias toward the prototype seat. However, it is assumed that any bias towards the motorcycle seat prototype would disappear through the riding duration if the seat did not truly adjust and maintain riding posture throughout the riding process. Due to time constraints (pandemic outbreak) and limited resources such as budget, it was not possible to include all aspects of the problem with a bigger sample size in this research. Besides that, there was only one prototype available for this research.

**Conclusion**

In summary, the present study proved that lumbar support with a built-in massager system successfully maintains and improves the spinal posture angle ergonomically throughout 20 minutes of riding. Although there is a positive outcome in this research, there is still a lack of evidence (i.e. muscle fatigue, discomfort, muscle activity) for this study. Hence, further study in the artificial laboratory and clinical settings as well as virtual testing is needed to support these findings.

**Acknowledgements**

The author would like to thank all of the people who were involved in this study especially the Royal Malaysian Police (RMP).

**References**

[1] Fernandes RD, da Silva Pataro SM, De Carvalho RB, Burdorf A. The concurrence of musculoskeletal pain and associated work-related factors: a cross sectional study. BMC Public Health. 2016 Dec;16(1):1-9.
[2] De Carvalho DE, Callaghan JP. Spine posture and discomfort during prolonged simulated driving with self-selected lumbar support prominence. Human factors. 2015 Sep;57(6):976-87.
[3] Makhsous M, Lin F, Hendrix RW, Hepler M, Zhang LQ. Sitting with adjustable ischial and back supports: biomechanical changes. Spine. 2003 Jun 1;28(11):1113-21.
[4] Pope MH, Goh KL, Magnusson ML. Spine ergonomics. Annual review of biomedical engineering. 2002 Aug;4(1):49-68
[5] Guo LX, Dong RC, Zhang M. Effect of lumbar support on seating comfort predicted by a whole human body-seat model. International Journal of Industrial Ergonomics. 2016 May 1;53:319-27.
[6] Sprigle S, Sonenblum SE, Feng C. Pressure redistributing in-seat movement activities by persons with spinal cord injury over multiple epochs. PloS one. 2019 Feb 13;14(2):e0210978.
[7] Wang SJ, Sommer B, Cheng W, Schreiber F. The Virtual-Spine Platform—Acquiring, visualizing, and analyzing individual sitting behavior. PloS one. 2018 Jun 13;13(6):e0195670.
[8] Karuppiah K, Salit MS, Ismail MY, Ismail N, Tamrin S. Evaluation of motorcyclist's discomfort during prolonged riding process with and without lumbar support. Anais da Academia Brasileira de Ciências. 2012 Dec;84(4):1169-88.
[9] Donnelly CJ, Callaghan JP, Durkin JL. The effect of an active lumbar system on the seating comfort of officers in police fleet vehicles. International journal of occupational safety and ergonomics. 2009 Jan 1;15(3):295-307.
[10] Durkin JL, Harvey A, Hughson RL, Callaghan JP. The effects of lumbar massage on muscle fatigue, muscle oxygenation, low back discomfort, and driver performance during prolonged driving. Ergonomics. 2006 Jan 15;49(1):28-44.
[11] Mansfield N, Sammonds G, Nguyen L. Driver discomfort in vehicle seats–Effect of changing road conditions and seat foam composition. Applied ergonomics. 2015 Sep 1;50:153-9.
[12] Rashid H, Omar AR, Ahmad IN, Mohamed Z, Wan Fauzi WM, Mahmud Z, Haron R. Motorcyclists’ Prolonged Riding Simulation: The Setup and Procedures. Journal of Mechanical Engineering (JMechE). 2017(5):197-208.
[13] Diyana MA, Karmegam K, Shamsul BM, Irniza R, Vivien H, Sivasankar S, Syahira MP, Kulanthayan KC. Risk factors analysis: Work-related musculoskeletal disorders among male traffic policemen using high-powered motorcycles. International Journal of Industrial Ergonomics. 2019 Nov 1;74:102863.
[14] Yusof M, Diyana NA. Association between occupational and non-occupational risk to work-related musculoskeletal disorders among traffic police riders (Doctoral dissertation, Master’s thesis]. Universiti Putra Malaysia).
[15] Gouvêa GR, Vieira WD, Paranhos LR, Bernardino ID, Bulgareli JV, Pereira AC. Assessment of the ergonomic risk from saddle and conventional seats in dentistry: A systematic review and meta-analysis. PloS one. 2018 Dec 17;13(12):e0208900.
[16] Carcone SM, Keir PJ. Effects of backrest design on biomechanics and comfort during seated work. Applied Ergonomics. 2007 Nov 1;38(6):755-64.
[17] Gong H, Sun L, Yang R, Pang J, Chen B, Qi R, Gu X, Zhang Y, Zhang TM. Changes of upright body posture in the sagittal plane of men and women occurring with aging–a cross sectional study. BMC geriatrics. 2019 Dec;19(1):1-1.
[18] Kolich M, Taboun SM, Mohamed AI. Low back muscle activity in an automobile seat with a lumbar massage system. International journal of occupational safety and ergonomics. 2000 Jan 1;6(1):113-28.
[19] Deros BM, Daruis DD, Nor MJ. Evaluation of car seat using reliable and valid vehicle seat discomfort survey. Industrial Engineering and Management Systems. 2009;8(2):121-30.
[20] Voinea GD, Butnariu S. Wearability assessment of an equipment for spine posture monitoring. In2017 E-Health and Bioengineering Conference (EHB) 2017 Jun 22 (pp. 25-28). IEEE.
[21] Mörl F, Bradl I. Lumbar posture and muscular activity while sitting during office work. Journal of Electromyography and Kinesiology. 2013 Apr 1;23(2):362-8.
[22] Ma’arof MI, Omar AR, Ahmad IN, Jaafar R, Fauzi WM, Rahim AA, Rashid H, Mahmud Z. The motorcycle design parameter database (MDPD) for different motorcycle models. Procedia Manufacturing. 2015 Jan 1;3:2581-8.
[23] Kwon Y, Kim JW, Heo JH, Jeon HM, Choi EB, Eom GM. The effect of sitting posture on the loads at cervico-thoracic and lumbosacral joints. Technology and Health Care. 2018 Jan 1;26(S1):409-18.
[24] Ma’arof MI, Omar AR, Haron R, Husain H, Wahab MA. Muscular Activities Measurements of Forward Lean and Upright Sitting Motorcycling Postures via Surface Electromyography (sEMG). InMATEC Web of Conferences 2017 (Vol. 135, p. 00046). EDP Sciences.
[25] Ceunen E, Zaman J, Vlaeyen JW, Dankaerts W, Van Diest I. Effect of seated trunk posture on eye blink startle and subjective experience: comparing flexion, neutral upright posture, and extension of spine. PloS one. 2014 Feb 7;9(2):e88482.
[26] Alyami H, Albarrati AM. Comparison of Spinal Angles in a Typing Task on a Laptop and a Desktop Computer: A Preliminary Study. American Journal of Occupational Therapy. 2016 Nov 1;70(6):7006350020p1-8.
[27] Shafiei UK, Karuppiah K, Tamrin SB, Meng GY, Rasdi I, Alias AN. The effectiveness of new model of motorcycle seat with built-in lumbar support. Jurnal Teknologi. 2015 Dec 20;77(27).
[28] Tanaka TH, Leisman G, Mori H, Nishijo K. The effect of massage on localized lumbar muscle fatigue. BMC complementary and alternative medicine. 2002 Dec;2(1):1-8.
[29] Van Poppel MN, Hooftman WE, Koes BW. An update of a systematic review of controlled clinical trials on the primary prevention of back pain at the workplace. Occupational Medicine. 2004 Aug 1;54(5):345-52.
[30] Franz M, Zenk R, Vink P, Hallbeck S. The effect of a lightweight massage system in a car seat on comfort and electromyogram. Journal of manipulative and physiological therapeutics. 2011 Feb 1;34(2):107-13.

**Supporting Information**

**S1 Table. Spinal Angle Profile Throughout 20 Minutes (N=24)**
