# Supplementary material for: Effectiveness of lumbar support with built-in massager system on spinal angle profiles among high-powered traffic police motorcycle riders: A randomised controlled trial
Source: PLoS One. 2021 Oct 19;16(10):e0258796. doi: 10.1371/journal.pone.0258796 (PMC8525748; doi:10.1371/journal.pone.0258796)
Supplement: Attachment — Submitted filename: Response to reviewer.docx [file pone.0258796.s005.docx]

Dear Dr Ahmed Mancy Mosa (Academic Editor),

Thank you for giving me the opportunity to submit a third revised draft of my manuscript titled ***effectiveness of lumbar support with built-in massager system on spinal angle profiles among high-powered traffic police motorcycle riders: A randomised controlled trial.*** We appreciate the time and effort that you and the reviewers have dedicated to providing your valuable feedback on my manuscript. We are grateful to the reviewers for their insightful comments on our paper. We have been able to incorporate changes to reflect most of the suggestions provided by the reviewers. We have highlighted the revisions within the manuscript.

Here is a point-by-point response to the reviewers’ comments and concerns.

| **No.** | **Comments from Reviewer 3** | **Author response** |
| --- | --- | --- |
| 1. | Thank you for responding to my comments. I believe the authors dedicated a lot of time going through all the reviews. Congrats on that! I still believe the authors did not explain the randomization method. Mentioning that randomization was performed is not enough, how was it done? They are several randomization methods? Which one did you use? | Firstly, we want to apologize because we are not clear on the type of randomization intended by the reviewer. However, this is the best explanation that we can give for this remark.  *A randomised controlled trial,* ***pretest-posttest control group design*** *was conducted among 24 traffic police riders who ride a high-powered motorcycle (Honda CBX 750).* ***A simple random sampling was used in this study which the*** *subjects were randomly assigned to the control group (12 riders) and experimental group (12 groups). Data collection commenced in March 2020 and finished in July 2020.*  Further explanation had been explained in line 138-165. |
| 2. | Also, the authors compared pre and post in exp and control group (fine); compared pre (cont) vs pre (int) and post (cont) vs (pos int), which is also fine but the most important comparison was not performed. The mean (median) difference between group is the one that is really informative and should be compared to the MCID. | Thank you for pointing this out. However, this MCID actually will be discussed in details in our future paper. The main objective for this work is to evaluate the effect of lumbar support with a built-in massager system on spinal angle profiles among traffic police riders which focus on the comparison of pretest-posttesst and between control-experimental groups. Nevertheless, for future studies, this will be a great insight and we will be sure to use it. |

| **No.** | **Comments from Reviewer 4** | **Author response** |
| --- | --- | --- |
| 1. | Reviewer #4: Abstract  Background  Research gap was not stated  method  major statistical analysis was not specified  Main body  major statistical analysis was not specified  results  The major statistical model output were not presented | We really appreciate your valuable comments here. We find it really useful to improve our manuscript. After discussion with our co-authors, we have improved our manuscript (in the 2^nd^ revision) based on the given comments as below: -  *i. The context and research gap of traffic police riders suffered low back pain, and MSD which no research has been done on the spinal riding posture during a motorcycle ride was explained in line 72-87.*  *ii. The context and research gap in which there is a lack of data on the effectiveness of this intervention seat, lumbar support with a built-in massager system, in an in-field setting (on-the-road) was explained in line 96-99.*  *The major statistical analysis used in this study was the Mann-Whitney test and Wilcoxon signed-ranked test. The details of the statistical analysis were explained in line 233-243.*  *The sampling technique was explained in Participant Recruitment (line 132-158).*  However, the similar comments are asked again in this 3^rd^ revision. Thus, we decided to explained this in the abstract since it has a word abstract in the beginning of the comments.  ***Traffic police riders are exposed to prolonged static postures causing significant angular deviation of the musculoskeletal, including the lumbar angle (L1-L5). This postural alteration contributes to awkward posture, musculoskeletal disorders and spinal injury, especially in the lower back area, as it is one of the most severe modern diseases nowadays****. Thus, the study aimed to evaluate the effect of lumbar support with a built-in massager system on spinal angle profiles among traffic police riders. A randomised controlled trial (pre-testpost-test control design) was used to assess spinal angle pattern while riding the high-powered motorcycle for 20 minutes. Twenty-four traffic police riders were randomly selected to participate and 12 riders were assigned to the control group and 12 riders to the experimental group. The pre-test and post-test were conducted at a one-week interval. Each participant was required to wear a TruPosture Smart Shirt (to monitor spinal posture). The TruPosture Apps recorded the spinal angle pattern. The data indicated that the police riders using motorcycle seat with lumbar support and built-in massager system showed a huge improvement in maintaining posture which only involves slight spinal angle deviation changes from the spinal reference angle throughout the 20 minutes ride.* ***The data collected then were analysed using the Mann-Whitney test and Wilcoxon signed-ranked test to verify a statistically significant difference between and within the control and experimental groups.*** *There were significant differences in all sensors between the control group and experimental groups (p<0.05) and within the experimental group. According to the findings, it can be said that the ergonomic intervention prototype (lumbar support with built-in massager system) successfully helps to maintain and improve the natural curve of the spinal posture. This indirectly would reduce the risk of developing musculoskeletal disorders and spinal injury among traffic police riders.*  We do provide the justification as requested as based on our understanding. Please do advise us in details if we need further revision as we feel your valuable comments will improve further our manuscript. Thank you. |
